# Synthesis and Perspectives of Oriented Growth of Double-Perovskite Cs_2_SnI_6_ in the Presence of Antimony

**DOI:** 10.3390/nano16090553

**Published:** 2026-04-30

**Authors:** Shodruz T. Umedov, Anastasia V. Grigorieva, Egor V. Latipov, Alexander V. Dzuban, Alexander V. Knotko, Andrei V. Shevelkov

**Affiliations:** 1 Department of Materials Science, Lomonosov Moscow State University, Leninskie Gory 1/73, 119991 Moscow, Russia; shodruzumedov@gmail.com (S.T.U.);; 2 Department of Chemistry, Lomonosov Moscow State University, Leninskie Gory 1/3, 119991 Moscow, Russia; 3 Institute of Nanotechnology of Microelectronics of the Russian Academy of Sciences, Leninskiy Prospekt, 32A, 119334 Moscow, Russia

**Keywords:** Cs_2_SnI_6_, lead-free halide perovskite, antimony, melt crystallization, oriented growth, optical absorption, deep defects

## Abstract

Vacancy-ordered double-perovskite Cs_2_SnI_6_ is known to be a good candidate for perovskite photovoltaics, as it is a light harvesting material which has potential both as an individual compound and as a component of a composite material. The compound is interesting due to being free of atom sites in B cationic positions, making the lattice “breathable” and giving it optoelectronic characteristics that vary with dopants. Here, antimony was examined as a possible heterovalent dopant with an ionic radius larger than that of Sn^4+^. In practice, it has been found that most of the materials are composites of Cs_2_SnI_6_ and Cs_3_Sb_2_I_9_ phases. In the CsI–SnI_4_–SbI_3_ phase triangle, the melt crystallization process produced a layered (111)-oriented microstructure of crystallites with an increasing percentage of antimony. Two-dimensional perovskite materials look more promising in the decomposition of a solid solution to Cs_2_SnI_6_ and Cs_3_Sb_2_I_9_ phases than in heterophase nucleation. The observed effect of (111)-oriented growth could be translated to other inorganic halides to form new oriented films or single crystals of perovskite materials. Diffuse reflectance spectroscopy showed an additional absorption shoulder in the NIR region for all groups of compounds, most likely induced by point defects in I sublattices of Cs_2_SnI_6_. Expanding the Cs_2_SnI_6_ absorption range to the NIR region could lead to new perspectives for its application.

## 1. Introduction

Air-stable and less toxic tin-based Cs_2_SnI_6_ with a direct band gap, which belongs to the K_2_PtCl_6_ type but is known also as vacancy-ordered double perovskite Cs_2_SnI_6_ [1], has demonstrated potential use in photovoltaics, optoelectronics, and some other applications. Recent theoretical studies have shown that solar cells based on cesium iodostannate (IV) can achieve a power conversion efficiency (PCE) of up to 27% [1,2,3,4]. In practice, power conversion efficiency varies around 1% since it is difficult to obtain continuous thin films of excellent quality and to choose optimal *p*-*n* charge carrier transport layers [5,6,7,8]. Various strategies for balancing carrier transport in perovskite devices are under discussion nowadays [9], making perovskite compounds attractive prospects for further investigation.

Xiao et al. [10], Mokurala et al. [11,12], and Khan et al. [13] studied the visible light photoelectrical response of Cs_2_SnI_6_ under various conditions and found great performance as a component of flexible and stretchable photodetectors. Also, it is noteworthy that composites based on Cs_2_SnI_6_ show a high piezoelectric response [14]. According to recent research in the field of photonics, Cs_2_SnI_6_ nanocrystals have shown unusual light-defocusing properties that can create new possibilities in nonlinear optics applications [15]. Cesium iodostannate (IV) also shows other excellent functional properties. Wang et al. have shown photocatalytic reduction of CO_2_ on the Cs_2_SnI_6_/SnS_2_ nanocomposite interface and have investigated photoelectrochemical performances of Cs_2_SnI_6_ [16]. Furthermore, the photocatalytic behavior of Cs_2_SnI_6_ in the photodecomposition of organic dyes such as methyl orange, methyl violet, methylene blue, and rhodamine B was investigated and discussed by Yousefzadeh et al. [17]. In addition, the Cs_2_SnI_6_ film’s mechanisms for room-temperature NO_2_ sensing were analyzed by Pham et al. [18].

Despite multiple possible application strategies, it still remains promising to tune or improve the electric and optical properties of cesium iodostannate (IV) via the formation of substitution solid solutions. A substitution of iodine in the anionic sublattice with other halogens, such as Br, Cl, or F, has been examined recently [7,19,20,21,22,23]. It is an unusual objective to substitute large Cs with a monovalent cation or substitute Sn with a cation with a different charge. The effect of Rb/Ag [8] and Li [24] doping of Cs_2_SnI_6_ on its optoelectronic properties, film morphology, and device photoelectric response has been studied recently. Another strategy is the heterovalent substitution of Sn in the Cs_2_SnI_6_ structure. The heterovalent substitution of Sn^4+^ with In^3+^ or Ga^3+^ cations and its effect on the microstructure and optoelectronic properties of the Cs_2_SnI_6_ phase has been considered in detail [25,26]. In most cases, the authors declare notable results for optical or transport properties of Cs_2_SnI_6_ but do not discuss the precise structure of solid solutions or their thermodynamic stability.

Nowadays, we are observing a “boom” in low-dimensional perovskite halides [27,28]. In addition to 2D and 1D lattices and micromorphology of such compounds, the anisotropy of physicochemical characteristics is also important for further practical applications. Smart interface engineering for classic or tandem optoelectronic devices also requires an investigation of intergrain boundaries and oriented crystallization [29,30]. The oriented growth of thin films and the formation of heterostructures of complex iodides are topics of discussion [31,32]. Here, we report on an original synthetic route to the oriented growth of an inorganic perovskite material, Cs_2_SnI_6_, and discuss perspectives on its oriented growth as a film.

Variations in elemental ratios in the CsI–SnI_4_–SbI_3_ ternary system were applied to analyze the phase compositions of materials. In the present system, Sb(III) is an additive with a higher ionic radius than that of tin(IV), making the formation of substitution solid solutions less favorable than in the case of gallium or indium [25,26]. Along with our previous findings, it is evident that heterovalent doping by Sb is not the principal reason for significant changes in both the microstructure and optical properties of the Cs_2_SnI_6_ phase.

## 2. Materials and Methods

### 2.1. Synthesis Methods

Tin (IV) and antimony (III) iodides were synthesized from the corresponding metals, namely tin (>99.9%, Ruschim, Russia, >99.9%), antimony (>99.9%, Redkiy Metall, Novosibirsk, Russia), and elemental iodine (I_2_, purum, Reachim, Moscow, Russia) in carbon tetrachloride (CCl_4_, purum, >99.93%, Component-reaktiv, Moscow, Russia). The synthesized iodides were purified before use by sublimation and studied with XRD.

To prepare compositions Cs_2+x_Sn_1−x_Sb_x_I_6_ (the CS1 series), Cs_2_Sn_1−x_Sb_x_I_6−x_ (the CS2 series), Cs_2+x_Sn_1−x_Sb_2x_I_6+3x_ (the CS3 series), and Cs_2−x_Sn_1−x_Sb_x_I_6−2x_ (the CS4 series) (where *x* = 0–1 for each section), stoichiometric amounts of CsI (99.9%, SigmaAldrich, St. Louis, MO, USA):SnI_4_:SbI_3_ were weighed using a semi-micro analytical balance GR-202 (A&D Company, Limited, Tokyo, Japan) at room temperature in air and placed in preliminary dried quartz vials. All weights and mass ratios of the precursors are given in Table S1 in the Supplementary Information (SI).

Ampoules were evacuated to a residual pressure of 0.02 Torr. The lower part of the ampoule (where the sample was located) was cooled with liquid nitrogen, and the vial was sealed off quickly. The sealed vials were heated at a rate of 0.2 °C/min up to 620 °C and then annealed at this temperature for 48 h. The ampoules were then cooled to room temperature slowly in a shutdown furnace, then opened and studied in ambient conditions.

The synthesis of a pure Cs_2_SnI_6_ phase was carried out at 300 °C to avoid the separation of precursors in ampoules because of their different volatilities. For other compositions, we observed crystallization from a melt if the ampoule had been heated up to 620 °C.

### 2.2. Characterization Methods

Powder X-Ray diffraction (PXRD) measurements were performed on a Rigaku D/MAX 2500 diffractometer (Rigaku, Tokyo, Japan) equipped with a rotating copper anode (Cu-Kα_1,2_ radiation, graphite monochromator) and operated at 45 kV and 250 mA, from 5 to 80° in 2*Θ*; the scanning rate was 3° min^−1^ at a step of 0.02°. The experimental data were analyzed using WinXPow 2.24 (STOE, Darmstadt, Germany) and Jana2006 (Institute of Physics, Prague, Czech Republic) software to determine the phase composition. The unit cell parameters were refined using the Le Bail method.

UV–vis diffuse reflectance spectra were collected using a UV–vis spectrometer Lambda 950 (PerkinElmer, Shelton, CT, USA). Measurements were performed in a spectral range of 350–2000 nm with a step scan of 0.5 nm at 298 K with a scanning rate of 1 nm s^−1^.

The microstructure and elemental composition of the samples were studied using a scanning electron microscope with a field emission source LEO SUPRA 50VP (LEO Carl Zeiss SMT Ltd., Oberkochen, Germany) equipped with an X/MAX X-ray energy dispersive detector (Oxford Instruments, High Wycombe, UK) at various magnification. The electrons’ accelerating voltage was 21 kV, with a working distance 14–15 mm.

Raman spectroscopy was performed using a Renishaw InViaQontor Raman microscope (Renishaw, Gloucestershire, UK) equipped with linearly polarized lasers of 785 nm and 532 nm, 1200 lines/mm diffraction grating and Leica ×50 objective (N.A. = 0.8). Raman spectra were recorded with 0.1 mW incident power and spot size ≈ 1.5 μm. The recording parameters were as follows: measurement range 33–800 cm^−1^, exposition time 10 s, and 15 spectra accumulations. For the measurements conducted using optical microscopy, particles or crystallites with a minimum lateral dimension exceeding 30 μm were selected. This selection was made to enhance heat dissipation during laser exposure and to minimize surface-related effects.

Thermal analysis was carried out on a DSC 204 F1 Phoenix^®^ differential scanning calorimeter (NETZSCH, Selb, Germany). The sample was ramped up from 25 to 280 °C with a heating rate of 10 °C/min under an argon flow of 70 mL/min in an aluminum crucible. The instrument was previously calibrated for temperatures and enthalpies of phase transitions of pure (99.999%) standard substances in compliance with ASTM Practices E967, E968, and E2253: cyclohexane, Hg, Ga, benzoic acid, In, Sn, Bi, Pb, Zn, and CsCl. The mean estimated temperature error was 0.2 °C.

## 3. Results

### 3.1. Compositions in the Phase Triangle CsI–SnI_4_–SbI_3_

**Figure 1:**
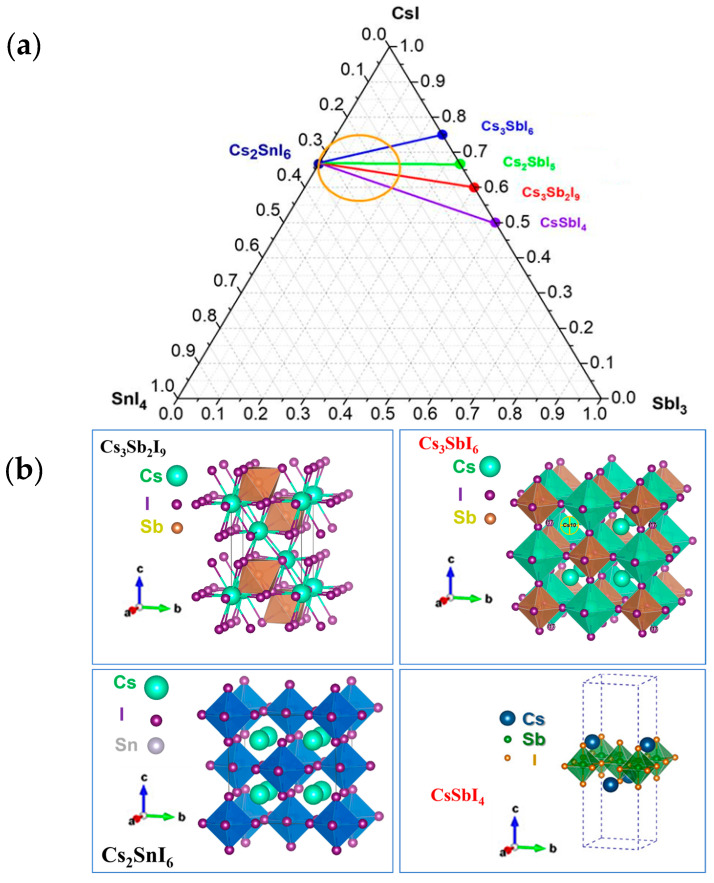
Gibbs triangle showing the positions of the studied samples in the CsI–SnI_4_–SbI_3_ ternary system (**a**), and (**b**) related crystal structures at four points on the CsI–SbI_3_ side according to the literature data [33,34].

Samples in four sections of the CsI:SnI_4_:SbI_3_ ternary system, namely, CS1, CS2, CS3, and CS4, were investigated according to the Gibbs triangle given in Figure 1a. The corresponding masses of precursors are given in Table S1 of the Supplementary Information (SI). It can be seen that for pure Cs_2_SnI_6_ (a molar rate of antimony, *x*, is 0), the sample obtained at 620 °C is not a single phase, and therefore we considered the diffraction pattern of Cs_2_SnI_6_ obtained at 300 °C (since it is a single-phase sample). The corresponding diffraction patterns for Cs_2_SnI_6_ compositions fused at 300 °C or 620 °C are given in Figure S1 of the SI for comparison. In the binary system CsI*–*SbI_3_, four theoretically predicted compounds could be expected, namely Cs_3_SbI_6_, Cs_2_SbI_5_, Cs_3_Sb_2_I_9_, and CsSbI_4_. Each composition is shown in the phase triangle, and the related unit cells of Cs_3_Sb_2_I_9_, Cs_3_SbI_6_ and CsSbI_4_ compounds are given in Figure 1b according to the literature [33,34].

**Figure 2:**
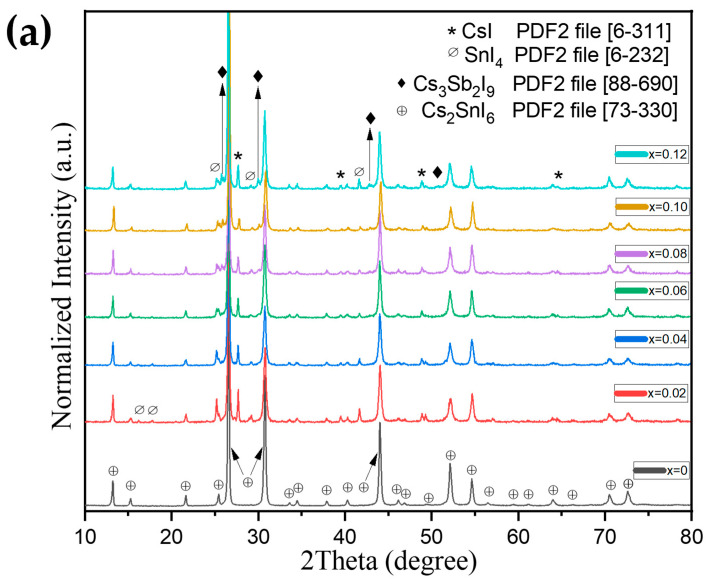

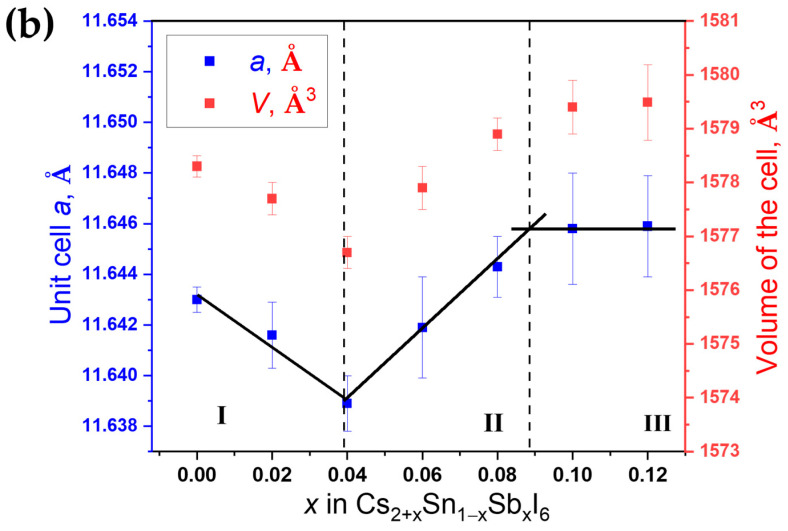
(**a**) XRD data of compositions Cs_2+x_Sn_1−x_Sb_x_I_6_ (*x* = 0–0.12) after fusing at 620 °C (the CS1 series); (**b**) Vegard’s plot showing the dependence of the lattice constant on the atomic composition of Sb in Cs_2_SnI_6_.

According to the XRD data, the samples of the Cs_2+x_Sn_1−x_Sb_x_I_6_ (CS1) series related to the Cs_2_SnI_6_–Cs_3_SbI_6_ line consist of the principal phase of Cs_2_SnI_6_ with a face-centered cubic structure (ICDD PDF2 file 73-330) (Figure 2a) (see Table S1 for the specific compositions). In addition to the main phase, there are also small amounts of impurity phases, namely cesium and tin(IV) iodides, CsI (ICDD PDF2 file 6-311) and SnI_4_ (ICDD PDF2 file 6-232), which served as precursors. On diffractograms for the samples with an antimony content of 6% (*x* = 0.06) or more, many strong and narrow reflections of the Cs_3_Sb_2_I_9_ phase (ICDD PDF2 file 88-690) can be seen.

Despite the high fusing temperature, there are no reflections of such impurity phases as oxides or oxoiodides of tin or antimony. Such impurities could be expected as results of the interaction of tin iodide with quartz ampoules and chemisorbed oxygen.

The unit cell parameter dependence on *x* in Vegard’s plot can be divided into three sections (Figure 2b). In the first section, we observe that as the percentage of antimony in the sample increases, the unit cell parameter decreases by up to 4 mol.% of Sb. As expected, due to the larger ionic radius of Sb^3+^ (0.76 Å) in comparison to Sn^4+^ (0.69 Å), the unit cell parameter, which we see in the second section of the graph, increases during the substitution. After ~9% of substitution, the unit cell parameter no longer changes, which possibly indicates the limit of the existence of the solid solution. It is also noticeable that the cell parameter is larger than for the Sb-free compound, which correlates with the higher ionic radius of Sb^3+^ in the octahedral position.

Analysis of the diffraction patterns shows that the relative intensity of many reflections of the Cs_2_SnI_6_ phase decreases as the percentage of antimony increases up to 4%; this holds true for the following reflections (h k l): (0 0 2); (1 1 7); (2 2 6). These planes pass through cesium and iodine atoms in the Cs_2_SnI_6_ lattice. We can therefore conclude that the reason for the decrease in the cell parameter may be the formation of iodine or cesium vacancies, which are fairly large atoms. It is also worth noting that, according to the literature data [35], iodine vacancies have a low value of formation energy, which is consistent with our conclusion.

For the Cs_2+x_Sn_1−x_Sb_x_I_6_ (CS1) series, starting from *x* = 0.02, the XRD patterns contain reflections (110), (200), (211), and (220) of cesium iodide, which was used as a precursor. The CsI reflections were used for zero correction for further calculations of Cs_2_SnI_6_ cell parameters in *x* = 0.02–0.12 CS1 samples. We observed that the relative intensities of the strong reflections (110) of CsI and (222) of Cs_2_(Sn,Sb)I_6_ do not change monotonically with the antimony content. This corresponds to the position of the CS1 section in the Gibbs triangle and confirms this section to be non-binary.

Also, none of the samples in the CS1 series contain the expected cubic tricesiumhexaiodoantimonate (the Cs_3_SbI_6_ phase) described in the literature [31]. Most likely, the Cs_3_SbI_6_ compound is not stable upon heating above 300 °C, and so all compositions of the CS1 series belong to the secondary phase triangle of Cs_2_SnI_6_–CsI–Cs_3_Sb_2_I_9_.

According to the phase analysis for the compositions at *x* = 1 (in each section), the samples consist of the Cs_3_Sb_2_I_9_ phase and CsI or SbI_3_, respectively, and include no other cesium iodoantimonates (III) described in the literature. The corresponding XRD results for the CS1 samples are given in Figure S2 in SI. It is obvious that the reflections of the Cs_3_Sb_2_I_9_ phase dominate in all diffractograms. The samples with *x* > 0.5 are not discussed below and not compared with the rest of the samples because the present discussion is focused on materials with the Cs_2_SnI_6_ structure only (Table 1).

The samples in the CS2 series belong to the Cs_2_SnI_6_–Cs_2_SbI_5_ line of the phase triangle CsI–SnI_4_–SbI_3_. For the CS2 samples, the annealing procedures were carried out at two temperatures: at 620 °C for melt crystallization and also at 300 °C to initiate solid-state sintering and to avoid the growth of large crystals of cesium iodoantimonate Cs_3_Sb_2_I_9_. We observed that the Cs_3_Sb_2_I_9_ phase was the only cesium iodoantimonate found in the samples. A reduction in the temperature of the calcinations down to 300 °C resulted in the presence of unreacted precursors, namely SnI_4_ and CsI, in the samples. At the same time, the relative intensity of reflections of cesium iodoantimonate Cs_3_Sb_2_I_9_ was much smaller for the samples fused at 300 °C, showing their lower crystallinity and higher percentage of binary iodides of cesium and tin (IV) used as precursors.

**Figure 3:**
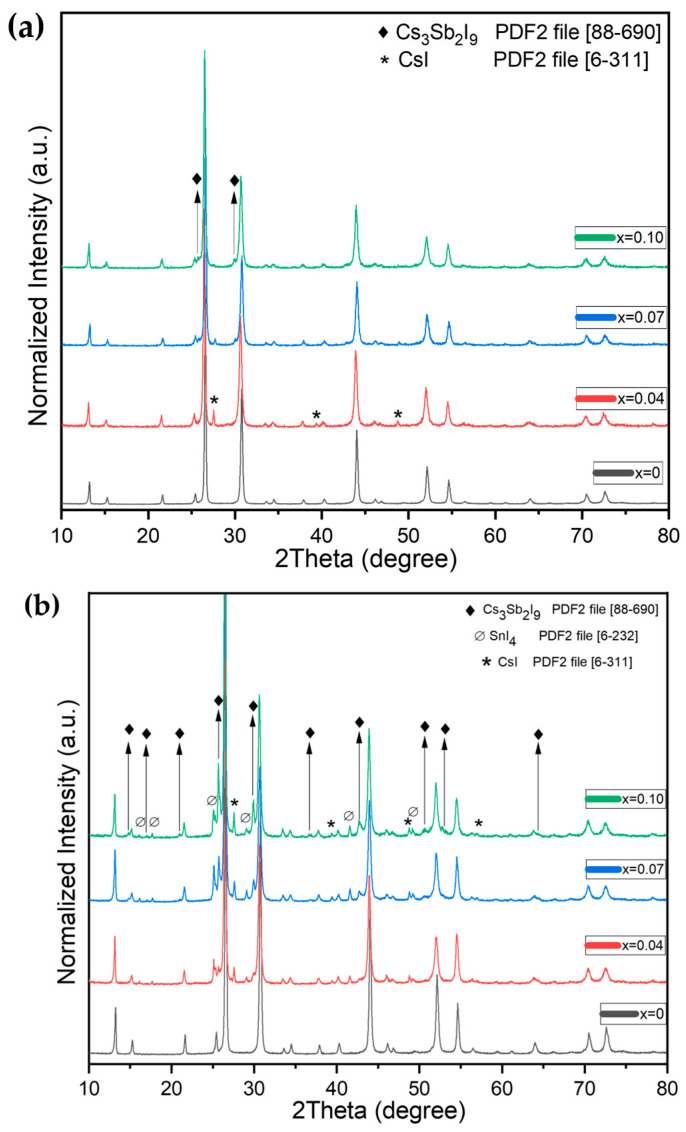

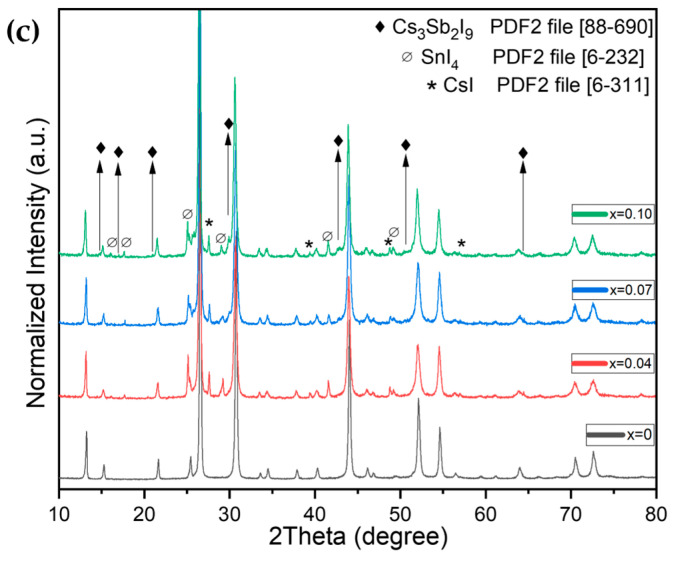
XRD analysis of other compositions sintered at 620 °C: (**a**) Cs_2_Sn_1−x_Sb_x_I_6−x_ (where *x* = 0–0.1) (the CS2 series), (**b**) Cs_2+x_Sn_1−x_Sb_2x_I_6+3x_ (where *x* = 0–0.1) (the CS3 series), and (**c**) Cs_2−x_Sn_1−x_Sb_x_I_6−2x_ (where *x* = 0–0.1) (the CS4 series).

Heating of the CS2 samples up to 620 °C leads to the melting of all three precursors. The resulting samples are single-piece and dark in color. The analysis of their phase composition shows the presence of two main phases, Cs_2_SnI_6_ and Cs_3_Sb_2_I_9_. Also, the admixture of CsI is found in the samples with *x* = 0.04 and 0.07 but disappears for *x* = 0.10 (Figure 3a). The sample with *x* = 0.10 shows no presence of CsI. The absence of crystalline cesium iodide could be related to the increase in the antimony content, resulting in the growing yield of the Cs_3_Sb_2_I_9_ crystals in the product. Reflections of the Cs_3_Sb_2_I_9_ phase are observed for *x* = 0.07 and 0.10 samples.

Similar trends with the phase composition are characteristic for Cs_2+x_Sn_1−x_Sb_2x_I_6+3x_ (the CS3 series) and Cs_2−x_Sn_1−x_Sb_x_I_6−2x_ (the CS4 series) samples on the Cs_2_SnI_6_–Cs_3_Sb_2_I_9_ and Cs_2_SnI_6_–CsSbI_4_ lines fused at 620 °C or 300 °C. All samples include unreacted precursors, namely, binary iodides CsI and SnI_4_, which did not react completely, especially, at a temperature of 300 °C. The corresponding diffractograms for the samples fused at 620 °C are shown in Figure 3b.

The products of the reactions were still Cs_2_SnI_6_ and Cs_3_Sb_2_I_9_, as was found for the CS1 and CS2 series. It is also noteworthy that in the case of the CS3 series, sharp reflections of SnI_4_ are rather narrow and intensive, probably as a result of a shift in the configuration points to the secondary phase triangle of Cs_2_SnI_6_–SnI_4_–Cs_3_Sb_2_I_9_ (Figure 1a). The corresponding points of the CS3 section belong to a binary section of stable compounds Cs_2_SnI_6_–Cs_3_Sb_2_I_9_. The demonstrated presence of the SnI_4_ phase could be a result of a partial segregation of compounds within vials.

In the case of the CS3 series, the XRD data show characteristic reflections of the Cs_3_Sb_2_I_9_ phase, which is the strongest one, and weak reflections of CsI. Thus, it is obvious from the XRD data presented above that all four series of the materials contain the main phase of iodostannate Cs_2_SnI_6_ and the only, most stable, iodoantimonate Cs_3_Sb_2_I_9_. The samples contain impurities of CsI and SnI_4_ for the CS1, CS3, and CS4 series and only CsI for the CS2 series, demonstrating partial separation of SnI_4_ in vials and its condensation in another zone. The effect of (311) reflections disappearing is comparatively lower than for the CS1 series.

Compared to the CS1 and CS2 series, the CS3 series shows rather high intensity and narrow reflections of tricesium iododiantimonate (III) Cs_3_Sb_2_I_9_ in the presence of SnI_4_ and CsI reflections, and a possible excess of SbI_3_ was not found in the samples. The presence of precursors could be associated with higher solidus temperatures in the Cs_2_SnI_6_–Cs_3_Sb_2_I_9_ section.

XRD data for the finely ground CS4 samples are presented in Figure 3c. For this series, we observed intensive reflections of the Cs_2_SnI_6_ iodide, Cs_3_Sb_2_I_9_ and the precursors cesium iodide and tin (IV) iodide the most. The reflections of Cs_3_Sb_2_I_9_ tricesium iododiantimonate (III) and other admixtures were rather weak. According to their phase compositions, the samples could be similar to those of the CS2 series, with an excess of SnI_4_.

### 3.2. The Microstructural Effect

**Figure 4:**
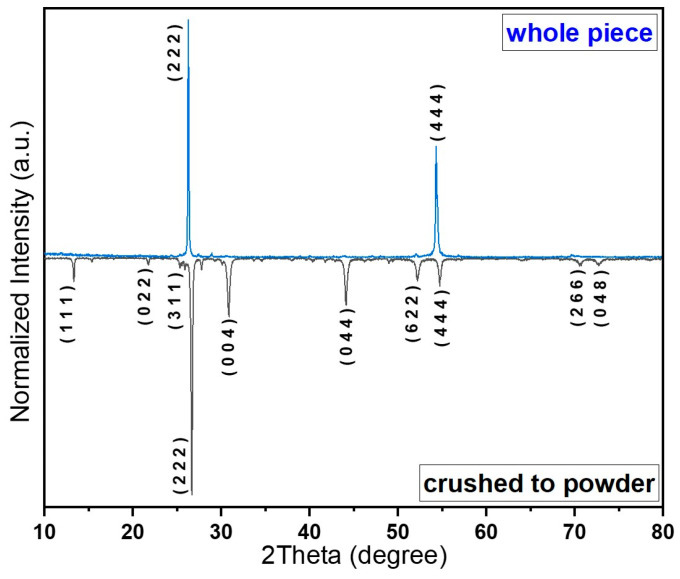
XRD data for the sample Cs_2+x_Sn_1−x_Sb_x_I_6_ with *x* = 0.1 before and after grinding (the CS1 series).

It is interesting that the CS1 samples demonstrate an exclusive microstructural effect, namely, as the antimony content increases, the intensity of some reflections of the Cs_2_SnI_6_ phase decreases. For instance, the (311) family is observed at 2*Θ* ~ 25.38° and the (622) at ~52.12°, with no overlapping with diffraction maxima from other phases. Starting from *x* = 0.04, the (311) reflections of the Cs_2_SnI_6_ phase disappear completely, which as we explained was a result of crystallographic texturing. The diffraction data for fine powders and single-piece samples of the Cs_2+x_Sn_1−x_Sb_x_I_6_ with *x* = 0.1 are given in Figure 4. The whole-piece sample of 0.5 cm was made from large crystals with a reflecting (111) plane.

**Figure 5:**
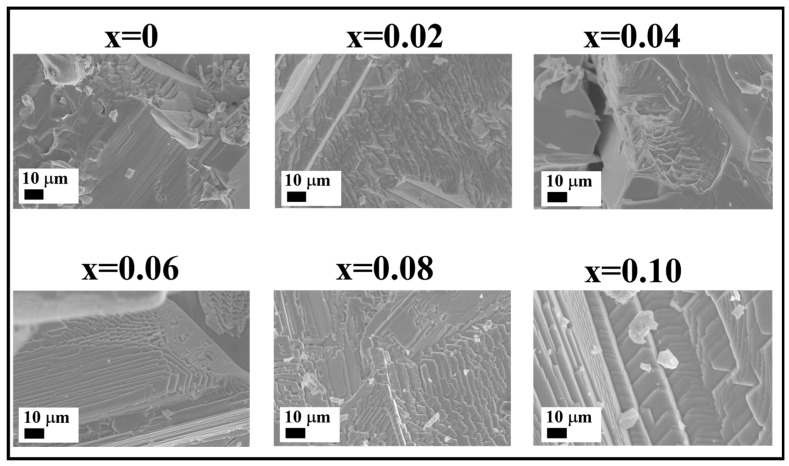
Fracture morphology SEM images of Cs_2+x_Sn_1−x_Sb_x_I_6_ (where *x* = 0–0.10) fused at 620 °C (the CS1 series).

Since the samples were fused at 620 °C, it would be interesting to discuss how their crystallization proceeds from a liquid phase (the melt) upon cooling in order to propose some approaches for further oriented growth of the double perovskite phase. For this purpose, unground pieces of samples from the fracture were separated for SEM and EDX measurements. The corresponding SEM micrographs for the CS1 series are given in Figure 5. Additional information is available in SI in Figure S3 for all four series.

The morphology of the CS1 series demonstrates the formation of large, oriented crystals with evident steps on crystal facets, especially for the samples with *x* of 0.04–0.1. The characteristic layered structure is typical for the following samples in the CS1 series, which include an excess of cesium with respect to tin and antimony.

The step growth microstructure is not that typical for the perovskite-like Cs_2_SnI_6_ iodide, and we see no similar morphology for the *x* = 0 sample that correlates with the literature [7,8]. The average thickness of individual steps in CS1 samples is about 200 nm. The thickness of the platelets does not diminish with Sb percentage, as could be seen in micrographs even with 2k× magnification. Edges of (111)-oriented crystallites with a hexagonal growth frontier can also be observed (see Figure 5, *x* = 0.06, 0.08 and 0.10). The steps at the crystal facets are observed for all six Cs_2+x_Sn_1−x_Sb_x_I_6_ (CS1) compositions (except *x* = 0), which shows some changes in the growth mechanism in the presence of Sb. On the other hand, the reflections of the CsI phase, starting from *x* = 0.02, demonstrate that an excess of cesium crystallizes as a single iodide and can hardly block the growth of perovskite crystals in the <111> direction.

We observed a dramatic change in the morphology of the CS1 samples from prismatic grains to layered structure, taking place at about 4 at.% of Sb in Cs_2+x_Sn_1−x_Sb_x_I_6_. The microstructural effect observed here could have different reasons. Most likely, it originates from the Cs_2_SnI_6_ lattice distortion, expected as a result of the formation of the substitution solid solution Cs_2+x_Sn_1−x_Sb_x_I_6_, where *x* = 0–0.04, as we observed a much more complex character of the evolution of cell parameter *a*. According to the discussion above (see Figure 2), the mixture of the saturated solid solution with CsI could be found in the range above 4 mol.% of Sb.

The second reason could originate from the different positions of configurative points in the composition triangle and the specifics of the crystallization process of the same Cs_2_SnI_6_ phase. Specifically, a decrease in solidus temperature in the presence of antimony iodide produces crystallization of Cs_2_SnI_6_ at the temperature of the ternary eutectic point T_E_ in the CsI–Cs_2_SnI_6_–Cs_3_Sb_2_I_9_ phase triangle. Crystallization of the Cs_2_SnI_6_ phase occurs in a wide temperature range up to T_E_, enabling more efficient growth of (111) planes and the overall evolution of crystal morphology to 2D-oriented crystals. Spinodal decomposition of the substitutional solid solution is also possible.

**Figure 6:**
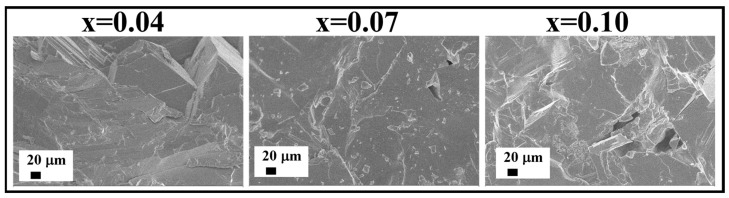
Fracture morphology SEM images of the Cs_2_Sn_1−x_Sb_x_I_6−x_ samples (*x* = 0.04–0.1) fused at 620 °C (the CS2 series).

It is noticeable that the effect of the reduction of the (311) and (622) reflections of the Cs_2_SnI_6_ phase described for the CS1 series above is not pertinent to the CS2 series (Figure 3). The relative intensities of diffraction maxima in the XRD patterns correspond to the literature data and demonstrate no texturing effect, as was observed for CS1 samples. According to the SEM data (Figure 6), the CS2 samples have a rather uniform microstructure. No oriented crystal growth or (111) texturing effects are observed for these samples, in contrast to the CS1 series. The crystals are above tens of microns; they are compact and have fewer intergrain boundaries than the CS1 samples. The growth mechanism for CS2 samples is not typical lateral growth, as was observed for the CS1 compositions with *x* = 0.06–0.10.

The elemental analysis (EDX) results are shown in Figures S4, S5, S6, S7 and S8 for CS1, CS2, CS3, and CS4, respectively. According to the EDX data, the stoichiometry ratios of cations in the CS4 samples correspond to the theoretical values (see SI, Figure S8). Specifically, the cation ratios and antimony percentage in the crystalline products differ from the initial ones only slightly, probably because of the low accuracy of the method or because of the micron-sized crystals of the corresponding iodostannate and iodoantimonate phases.

### 3.3. The Raman Spectroscopy of CS1 Samples

**Figure 7:**
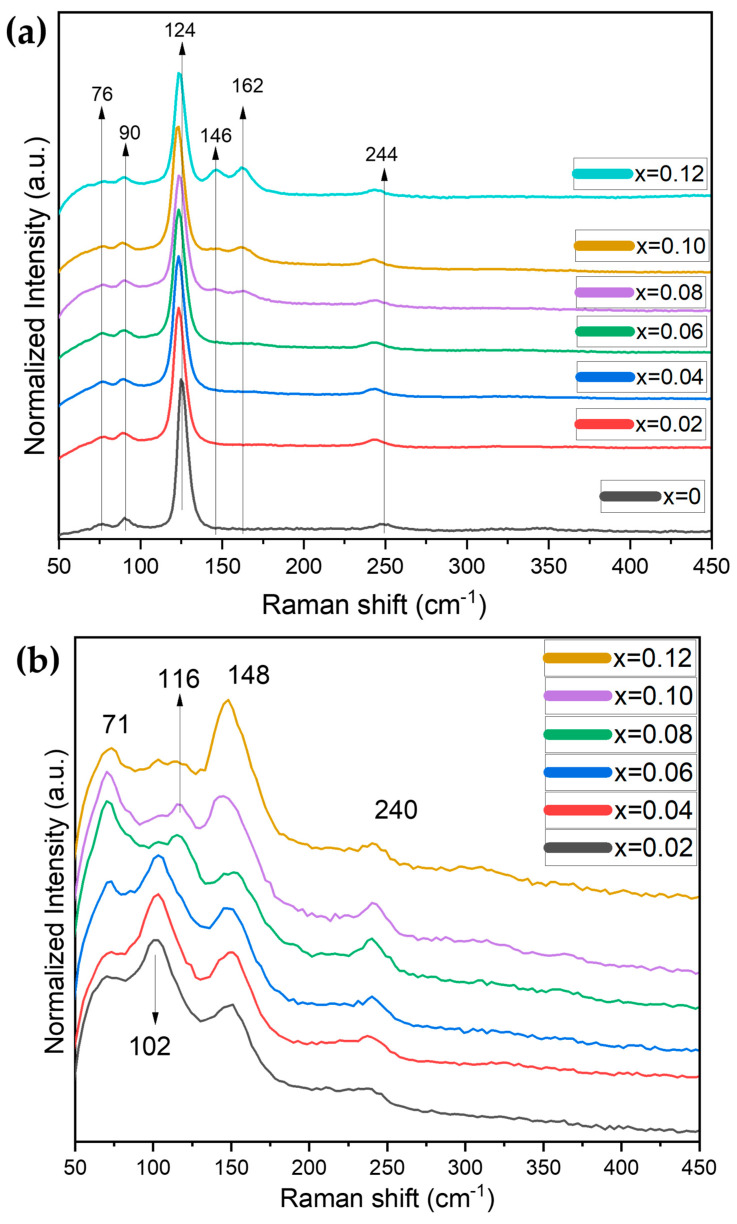
Raman spectra for the samples of the theoretical composition Cs_2+x_Sn_1−x_Sb_x_I_6_ (where *x* = 0–0.12) sintered at 620 °C (the CS1 series). The excitation laser was (**a**) 785 nm or (**b**) 532 nm.

Additionally, the crystal structure of the CS1 series has been characterized by Raman spectroscopy. As demonstrated by Figure 7a, the general vibration modes at ~76, 90, and 126 cm^−1^ could be related to collective vibrations in [SnI_6_]^2−^ octahedra in Cs_2_SnI_6_, namely, δ(F_2g_) mode at ~76 cm^−1^, ν(E_g_) at ~90 cm^−1^, and ν(A_1g_) at ~126 cm^−1^ with its second harmonic mode at ~249 cm^−1^ [36]. The characteristic stretching modes at 124 cm^−1^ and 248 cm^−1^ demonstrate an evident decrease in Raman shift to 122 cm^−1^ and 244 cm^−1^ in the presence of 2 mol.% of Sb or higher percentages, probably as a result of the increase in Sn–I bond lengths.

Also, with an increase in antimony percentage, two additional modes appear at about 146 cm^−1^ and 162 cm^−1^ (*x* > 0.06) which very probably belong to polyhedrons of Cs_3_Sb_2_I_9_. According to the literature [37,38], such vibration modes could originate from Sb–I vibration in the tetrahedral [SbI_4_]^−^ anion. According to [39,40], these two modes could be related to Sb–I asymmetric and symmetric stretching modes in a [Sb_2_I_9_]^3−^ bioctahedral complex with a small distortion. A direct comparison with the Raman spectrum for polycrystalline Cs_3_Sb_2_I_9_ shows that the observed vibration at 146 cm^−1^ corresponds well to the literature [41], while the vibration 162 cm^−1^ differs significantly. The first assumption therefore points to the presence of an admixture of antimony iodide which was not found by the XRD analysis. The second version is more optimistic because it relates to the insertion or substitution of Sb ions into the lattice of Cs_2_SnI_6_.

Figure 7b shows a series of Raman spectra collected under 532 nm laser excitation. These spectra differ significantly from the ones presented in Figure 7a, revealing a dramatic growth of 147 cm^−1^ and 162 cm^−1^ modes (as a shoulder) in comparison to the 73 cm^−1^, 89 cm^−1^, and 240 cm^−1^ modes of Cs_2_SnI_6_. At the same time, its most intensive characteristic mode A_1g_ at 123–126 cm^−1^ degrades significantly because of the decomposition of cesium hexaiodostannate (IV) under the laser beam or because of the microstructural effect discussed above. The characteristic modes of the Cs_3_Sb_2_I_9_ phase become stronger than the vibrations of Cs_2_SnI_6_.

**Figure 8:**
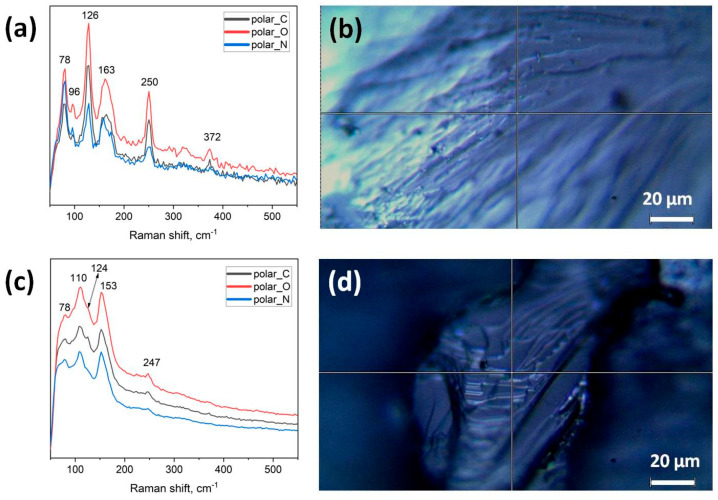
Raman spectra (532 nm excitation) of Cs_2+x_Sn_1−x_Sb_x_I_6_ (*x* = 0.10) sintered at 620 °C (CS1 series): (**a**,**c**) Raman spectra of the powdered sample and the whole-piece (111)-oriented sample, respectively (the latter rec-orded under C, O, and N polarizations); (**b**,**d**) optical images of the whole-piece sample in two crystallographic orientations.

For the CS1 series, we carried out the Raman spectroscopy study using both powdered samples and a whole-piece sample with the (111)-oriented microstructure. In the Raman spectra of the powder sample, we see the same characteristic modes of Cs_2_SnI_6_; namely, the δ(F_2g_) mode at ~78 cm^−1^, ν(E_g_) at ~96 cm^−1^, and ν(A_1g_) with its second and third harmonic modes (Figure 8a). For the whole-piece sample, the Raman spectra were recorded under three polarization configurations: circular (C), orthogonal (O), and normal (N). In Figure 8b,d, we see the single-piece sample with different crystallographic orientations. We also see in the corresponding Raman spectra a shift and a degradation of ν(A_1g_) mode and its harmonics; the ν(A_1g_) mode is observed at ~124 or 126 cm^−1^ and its second harmonic at ~247 or 250 cm^−1^. Notably, an additional feature at ~124 cm-1 appears in the C and O configurations but is absent in N, indicating anisotropic vibrational response of the oriented microstructure. This result confirms our previous conclusion about the oriented growth.

### 3.4. Spectral Characteristics of the Fine Powder Samples

**Figure 9:**
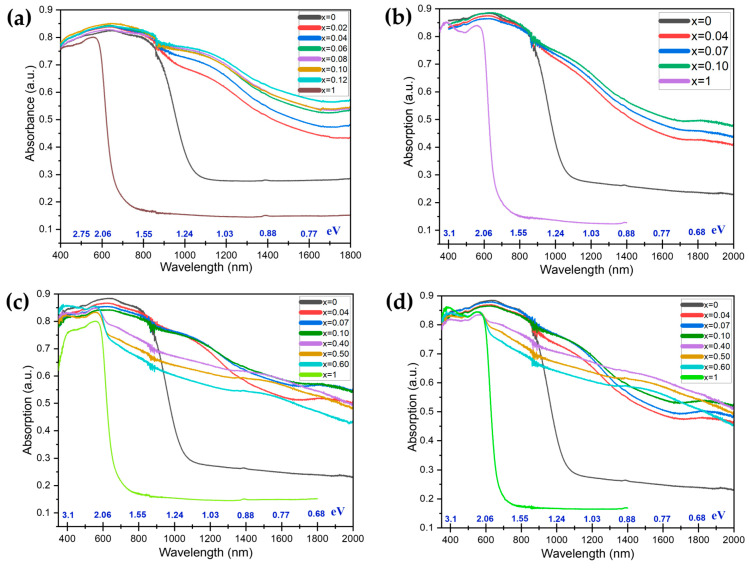
Absorption spectra of (**a**) Cs_2+x_Sn_1−x_Sb_x_I_6_ (where *x* = 0–1) (CS1), (**b**) Cs_2_Sn_1−x_Sb_x_I_6−x_ (where *x* = 0–1) (CS2), (**c**) Cs_2+x_Sn_1−x_Sb_2x_I_6+3x_ (where *x* = 0–1) (CS3), and (**d**) Cs_2−x_Sn_1−x_Sb_x_I_6−2x_ (where *x* = 0–1) (CS4). In the figures, the internal scale shows the wavelength values in the corresponding energy with an accuracy of two decimal places.

The finely ground powders of the samples with *x* = 0–0.12 from all four series were black in color. The optical absorption spectra of the pure phase Cs_2_SnI_6_ and composites are given in Figure 9. All absorption spectra of the composite samples differed from that of Cs_2_SnI_6_, which manifests in the appearance of two new absorption maxima or shoulders. The first one is a less intense peak at about 1900 nm (~0.65 eV). The second and more intense one is at 1150–1200 nm (~1.05 eV). As the content of antimony in the samples increases, the intensity of new peaks increases too, while the edges of the absorption spectra shift to the infrared region (“red shift”).

The observed UV–vis–NIR spectra show the growth of two additional maxima in the DRS with the percentage of antimony iodide. The intensity of the ~1.05 eV shoulder grows with the antimony percentage up to *x* = 0.06–0.07 for the CS1, CS3, and CS4 series and a little more (up to x = 0.10) in the case of the CS2 series, which demonstrates less anisotropy than the others.

The new absorption maxima revealed for the composite samples in all four series of the samples above could be related to a new group of defects in the Cs_2_SnI_6_ crystal structure. The optical and IR spectra of pure phases of precursors and cesium iodoantimonate (III) Cs_3_Sb_2_I_9_ have no principal electron transitions at 0.65 eV and 1.05 eV, which are evident for the two-phase samples with *x* = 0.4, 0.5 and 0.6.

Therefore, the appearance of two new absorption maxima can be explained by the formation of new defects or by the formation of additional self-defects known in the literature [1] for the pure phase Cs_2_SnI_6_. The corresponding electron relaxation energies observed from the diffuse reflectance spectra (DRS) in the NIR region should be compared to the theoretical self-defects in the Cs_2_SnI_6_ phase. According to Xiao et al. [35], energy values in the region of 0.6–1.2 eV could be related to V_I_ (0/+1), Sn_i_ (+2/+1 or 0/+1), and more rare defects such as Sn_Cs_ (0/+1), Cs_Sn_ (−1/0 and 0/+1), and I_i_ (−1/0). The most commonly expected kind of defect is a vacancy of iodine that is very typical for cesium iodostannate (IV) [1]. An increase in the concentration of iodine vacancies provides an increase in the carrier concentration and electrical conductivity of the material.

Deep defects in Cs_2_SnI_6_ could be suppressed by the I-rich atmosphere [22,23,24,40]. In the present study, the samples were obtained in stoichiometric ratios of compounds. It is most likely that the observed absorption edges and maxima in the NIR region correspond to deep defects which those concentrations stop growing, providing (111)-oriented growth of the Cs_2_SnI_6_ phase. If Cs_2_SnI_6_ is doped with a heterovalent cation M^3+^ and forms a substitutional solid solution, there should be a lack of iodine vapors in the vials [26]. The same is anticipated if there are surface groups of Sb–I octahedrons or heterophase nucleation of Cs_3_Sb_2_I_9_, as demonstrated above by Raman spectroscopy. It is also noticeable that we observed a saturation of the deep defect concentration above 4 at.% of Sb.

The optical bandgap value *Eg* for the Cs_2_SnI_6_ sample, estimated from the corresponding Tauc plot, is about 1.22 eV, which differs from the literature. The decreased value of *E_g_* could be a result of a significant Urbach tail, which makes an unreal correct estimation of *E_g_* for all other samples.

The calculated Urbach energy *E_U_* for the CS1 samples increases with the percentage of Sb up to *x* = 0.12 (see Table 2). The last one confirms that the two growth peaks in the NIR region could be associated not only with point defects in the Cs_2_SnI_6_ phase but also with surface-related defects (like dislocations and grain boundaries) in materials. The most significant increase in the *E_U_* is observed for *x* = 0.02 and *x* = 0.08.

## 4. Discussion

The analysis of XRD and SEM data for the CS1 series shows the change in micromorphology from more normal to (111)-oriented for a percentage of antimony above 4 at.%. The formation of substitutional solid solutions up to 4 at.% of Sb is still controversial, and the stoichiometric composition is still unknown. The corresponding XRD data give much less reliable results on the unit cell refinement than expected because the inaccuracy of the calculation is rather high (see Table 1). At the same time, for all four series of the samples, a small decrease in the unit cell parameter *a* for the samples with a low Sb percentage (*x* of 0.04 or below) was observed. This could be explained by the solid solution formation in this range. It is also evident that the Sb^3+^ cation is a perfect candidate to be substituted for the large Sn^2+^ cation in the corresponding perovskite CsSnI_3_, but is rather large for the smaller Sn^4+^ cation in octahedral positions in the crystal structure of Cs_2_SnI_6_.

The mechanism of the (111)-oriented growth of the Cs_2_SnI_6_ phase could be related to heterogeneous nucleation of the Cs_3_Sb_2_I_9_ phase at the perovskite crystallites in the melt or to spinodal phase decomposition of the solid solution. At the same time, we know that the Cs_3_Sb_2_I_9_ compound is a thermodynamically stable phase with a high melting temperature of above 600 °C. For comparison, the decomposition point of the perovskite Cs_2_SnI_6_ phase is closer to 300 °C [42]. Therefore, spinodal decomposition of the Cs_2+x_Sn_1−x_Sb_x_I_6_ solid solutions is a more probable mechanism. Such decomposition could take place at *x* = 0.06 and above, producing (111)-oriented crystallites of the cubic phase Cs_2_SnI_6_.

Regarding the UV–vis spectroscopy data, we very probably observe the growth of concentration of self-point defects in the iodine sublattice and the further formation of surface-related defects with an increase in Sb percentage. At *x* = 0.06, the increase in the defect concentration at higher Sb percentages was a result of phase decomposition.

The spinodal decomposition theory is also in accordance with the EDS data given in SI (see Figures S4–S8) for *x* = 0.01, 0.03, and other compounds. Specifically, the EDS data demonstrate a uniform distribution of Sb for the CS1 samples. In the case of heterophase nucleation, we should observe a less uniform elemental distribution of the antimony.

**Figure 10:**
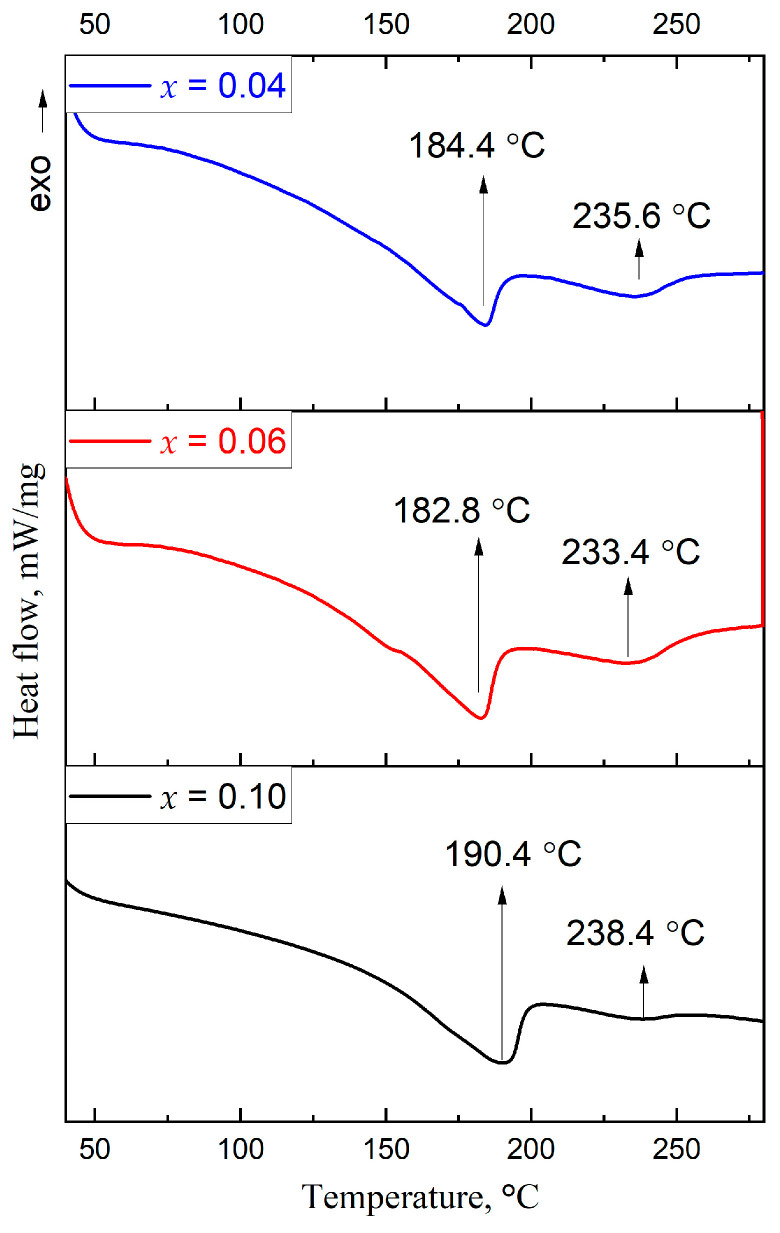
DSC data for Cs_2+x_Sn_1−x_Sb_x_I_6_ compositions where *x* = 0.04, 0.06 and 0.10 (the CS1 series).

The DSC data (Figure 10) for the *x* = 0.04, 0.06, and 0.10 samples in the CS1 series show two very broad endothermic peaks at 182.8–190.4 °C and 233.4–238.4 °C. At temperatures above 280 °C, complete evaporation of the sample takes place. The lower temperatures could be related to solidus temperatures and the higher ones to multiple liquidus points which are characteristic for ternary systems. The observed phase transition temperatures differ from the TG-DTA data reported for the pure Cs_2_SnI_6_ phase elsewhere, for which the melting point is above 300 °C [42]. It is probable that the CS1 samples correspond to a mixture of three or more compounds (as shown by the XRD data of the samples) with a eutectic temperature of 182–190 °C. Also, we observed the highest solidus temperature of for the *x* = 0.10 sample. This emphasizes the higher thermodynamic stability of the composite samples with (111)-oriented microstructure in comparison with the samples with lower Sb content.

The practical result of the investigation of Cs_2_SnI_6_-based samples is the observation of two absorption maxima in the NIR region for the two-phase samples of Cs_2_SnI_6_ and Cs_3_Sb_2_I_9_ and the samples of low percentages of the Cs_3_Sb_2_I_9_ phase (below 10 mol.%) or single-phase according to the XRD data (for *x* < 0.04). The phenomenon is interesting for near-infrared emitting scintillators and photocatalysts. The present results for the model perovskite-like iodide are disputable but could be extended to other complex iodide systems.

## 5. Conclusions

The (111)-oriented microstructure of the Cs_2_SnI_6_ phase depends on the presence of antimony and could most probably be observed if any other additives are used, as it is observed for organic additives, and provides 2D growth of perovskites instead of 3D. The mechanism of the (111)-oriented structure formation is not entirely clear, but we suggest that the heterophase nucleation of cesium iodoantimonate on the Cs_2_SnI_6_ grains is complicated when spinodal decomposition of the solid solution takes place. At the same time, the presence of Sb^3+^ in the reactionary melt results in deep defect levels in the NIR spectra of Cs_2_SnI_6_ that could expand the applications of the material.

## Figures and Tables

**Table 1:** The refinement of the unit cell parameter for the Cs_2_SnI_6_ phase with the Fm-3m cubic structure.

x	a, Å	V, Å^3^	Figure of Merit
The pure phase Cs_2_SnI_6_			
0	11.6430 (1)	1578.3 (2)	165
The CS1 series			
0.02	11.6416 (1)	1577.7 (3)	79
0.04	11.6389 (1)	1576.7 (3)	92
0.06	11.6419 (1)	1577.9 (4)	89
0.08	11.6443 (1)	1578.9 (3)	80
0.10	11.6466 (2)	1579.8 (5)	55

**Table 2:** The Urbach energy calculated from the absorption spectra of Cs_2+x_Sn_1−x_Sb_x_I_6_ (where *x* = 0–1) (the CS1 series). The sample *x* = 1 corresponds to the sample of total composition Cs_3_SbI_6_.

*x*	0	0.02	0.04	0.06	0.08	0.10	0.12	1
*E_U_*, meV	210	833	868	908	1069	1148	1225	188

## Data Availability

The data presented in this study are available in the article or Supplementary Materials.

## Supplementary Materials

The following supporting information can be downloaded at https://www.mdpi.com/article/10.3390/nano16090553/s1, Table S1: Weights of precursors for samples. The weighing error did not exceed ±0.0005 g in all measurements; Figure S1: PXRD data matching of a pure Cs_2_SnI_6_ composition sintered at 300 °C and 620 °C, respectively; Figure S2: PXRD data of Cs_2+x_Sn_1−x_Sb_x_I_6_ (*x* = 0.9–1) compositions (CS1 series); Figure S3: Images of fracture of CS1, CS2, CS3, and CS4 series at *x* = 0.1 compared to *x* = 0; Figure S4: Results of EDX analysis of samples Cs_2+x_Sn_1−x_Sb_x_I_6_ (where *x* = 0–0.04) (CS1series, part 1); Figure S5: Results of EDX analysis of samples Cs_2+x_Sn_1−x_Sb_x_I_6_ (where *x* = 0.06–0.12) (CS1 series, part 2); Figure S6: Results of EDX analysis of samples Cs_2_Sn_1−x_Sb_x_I_6−x_ (where *x* = 0.04–0.10) (CS2 series); Figure S7: Results of EDX analysis of samples Cs_2+x_Sn_1−x_Sb_2x_I_6+3x_ (where *x* = 0.04–0.10) (CS3 series); Figure S8: Results of EDX analysis of samples Cs_2−x_Sn_1−x_Sb_x_I_6−2x_ (where *x* = 0.04–0.10) (CS4 series); Figure S9: Optical photo of the sample x = 0.08 (CS1 series).
