# Supplementary material for: Synthesis and Perspectives of Oriented Growth of Double-Perovskite Cs2SnI6 in the Presence of Antimony
Source: Nanomaterials (Basel). 2026 Apr 30;16(9):553. doi: 10.3390/nano16090553 (PMC13165323; doi:10.3390/nano16090553)
Supplement: Supplementary file 1 [file nanomaterials-16-00553-s001.zip › nanomaterials-4241143-supplementary.pdf]

Supplementary Information

Synthesis and perspectives of oriented growth of double-perovskite Cs_2SnI_6 in the presence of antimony

Shodruz T. Umedov [1], Anastasia V. Grigorieva [1,2,*], Egor V. Latipov [3], Alexander V. Dzuban [2], Alexander V. Knotko [1], Andrei V. Shevelkov [1,2]

[1] Department of Materials Science, Lomonosov Moscow State University, Leninskie gory 1/73, 119991 Mocow, Russia.
[2] Department of Chemistry, Lomonosov Moscow State University, Leninskie gory 1/3, 119991 Mocow, Russia
[3] Institute of Nanotechnology of Microelectronics of the Russian Academy of Sciences, Leninskiy Prospekt, 32A, 119334 Moscow, Russia
* Correspondence: anastasia@inorg.chem.msu.ru;

Tables

Table S1. Weights of precursors for samples. The weighing error did not exceed ±0.0005 g in all measurements.

Sample series	*x*	CsI (g)	SnI_4 (g)	SbI_3 (g)
CS1	0	0.4534	0.5466	0
	0.02	0.4569	0.5344	0.0087
	0.04	0.4603	0.5222	0.0175
	0.06	0.4637	0.5101	0.0261
	0.08	0.4671	0.4981	0.0348
	0.1	0.4705	0.4861	0.0433
	0.12	0.4739	0.4742	0.0519
CS2	0.04	0.4554	0.527	0.0176
	0.07	0.4569	0.5122	0.0309
	0.1	0.4584	0.4973	0.0443
CS3	0.04	0.4524	0.5133	0.0343
	0.07	0.4517	0.4892	0.0591
	0.1	0.451	0.4659	0.0831
CS4	0.04	0.4504	0.5318	0.0178
	0.07	0.4481	0.5205	0.0314
	0.1	0.4457	0.5089	0.0454

Figures and Captions

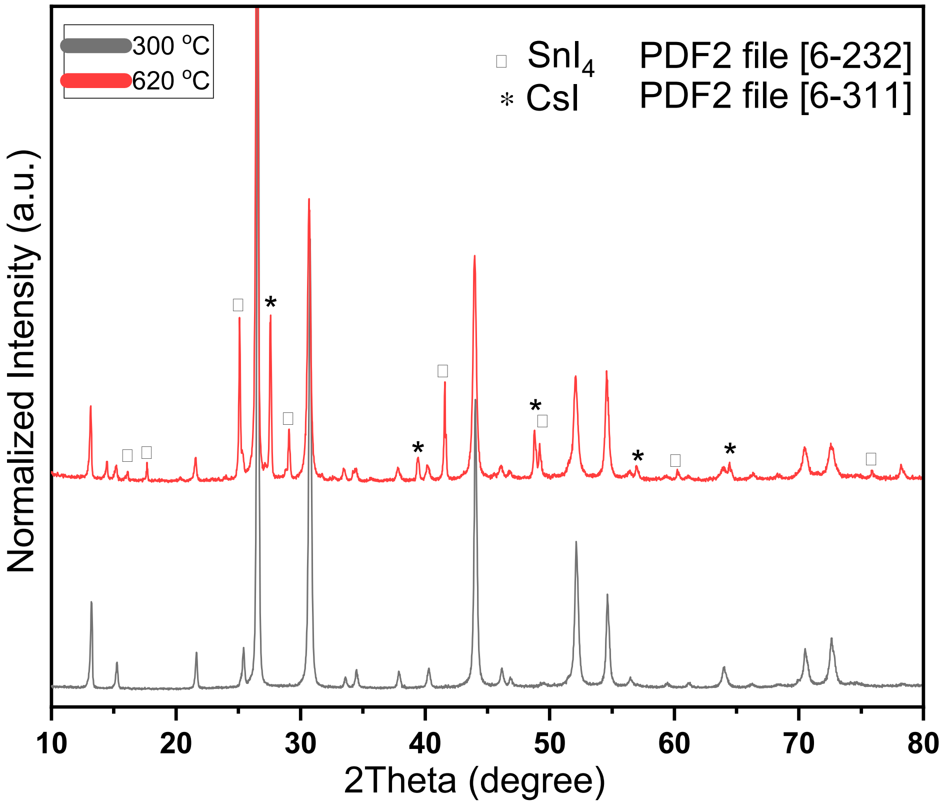

Figure S1. XRD data matching of a pure Cs_2SnI_6 composition sintered at 300°C and 620°C, respectively.

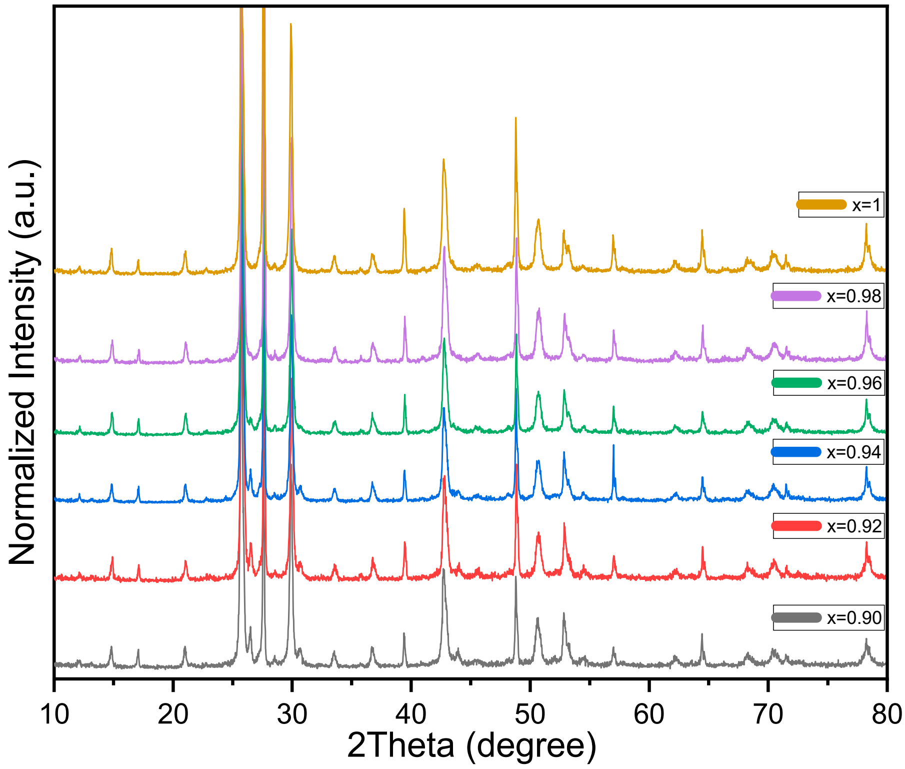

Figure S2. XRD data of $Cs_{2+x}Sn_{1-x}Sb_xI_6$ (x=0.9 – 1) compositions (CS1 series).

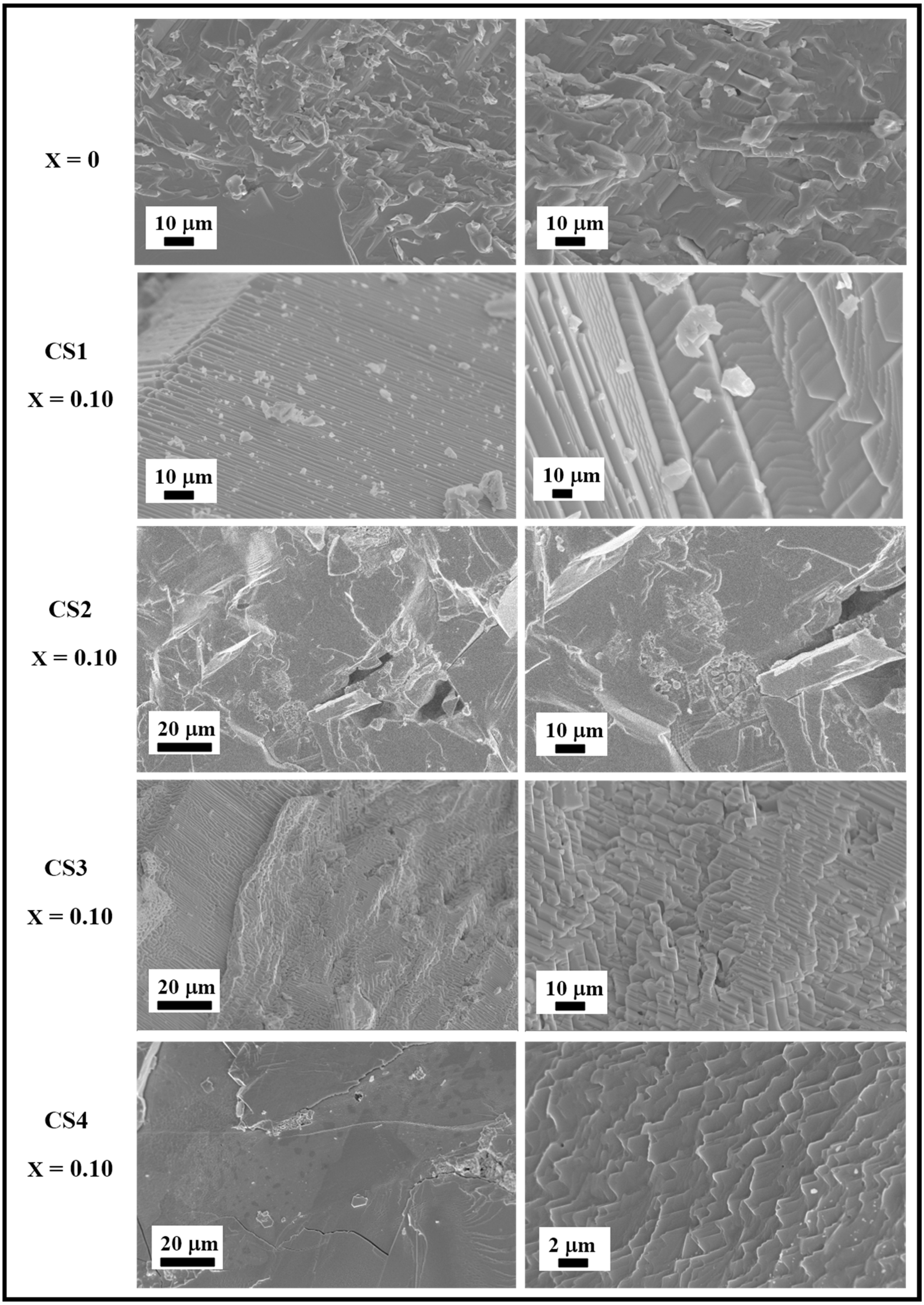

Figure S3. SEM images of fracture of CS1, CS2, CS3 and CS4 series at $x = 0.1$ compared to $x = 0$.

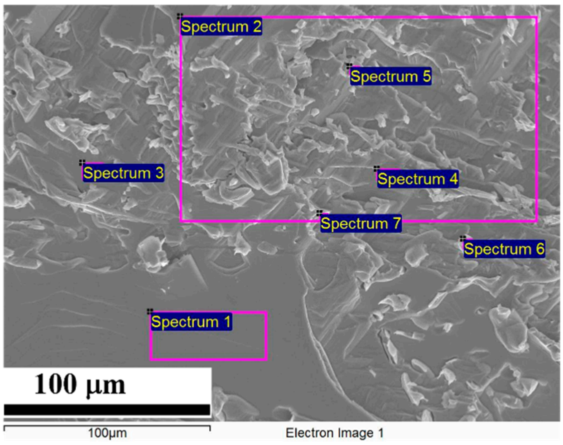

X = 0

	Sn	I	Cs	
Spectrum 1	11.67	67.68	20.66	
Spectrum 2	9.53	66.10	24.37	
Spectrum 3	11.05	67.07	21.87	
Spectrum 4	11.55	67.13	21.32	
Spectrum 5	9.15	65.09	25.76	
Spectrum 6	9.16	65.70	25.13	
Spectrum 7	8.74	63.90	27.36	

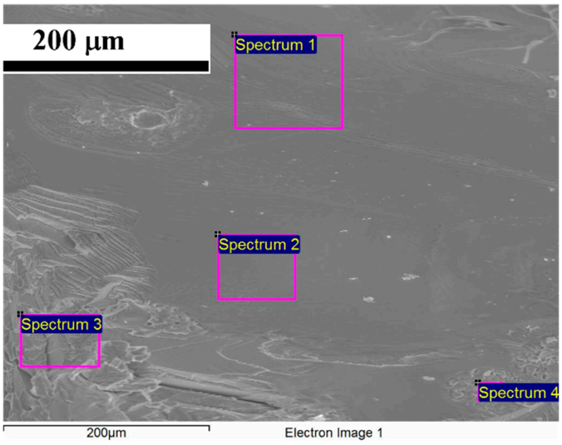

X = 0.01

	Sn	Sb	I	Cs
Spectrum 1	10.85	0.34	68.69	20.12
Spectrum 2	10.47	-	68.92	20.60
Spectrum 3	10.72	0.04	68.12	21.12
Spectrum 4	10.19	0.10	69.27	20.44

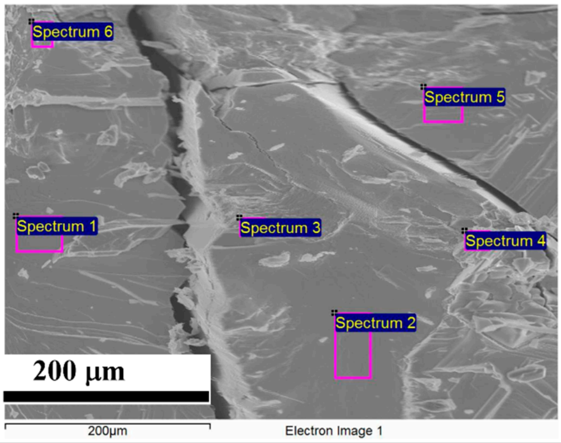

X = 0.03

	Sn	Sb	I	Cs
Spectrum 1	10.83	0.51	68.20	20.46
Spectrum 2	10.35	0.59	68.78	20.27
Spectrum 3	9.98	0.67	70.07	19.28
Spectrum 4	9.78	0.3	64.68	25.25
Spectrum 5	11.42	1.17	67.71	19.70
Spectrum 6	10.87	0.67	68.23	20.24

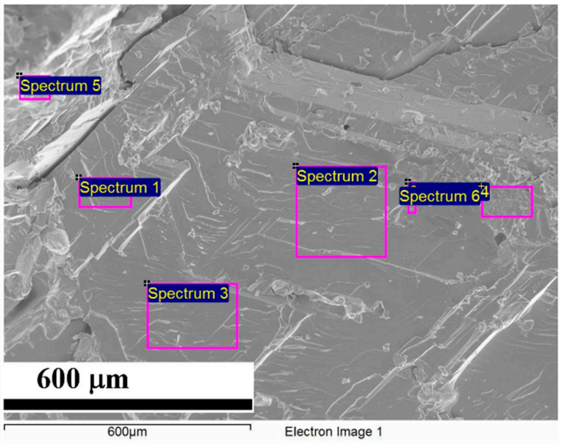

X = 0.04

	Sn	Sb	I	Cs
Spectrum 1	11.17	0.45	67.68	20.70
Spectrum 2	11.23	0.40	67.68	20.69
Spectrum 3	11.49	0.64	67.25	20.61
Spectrum 4	11.95	0.35	67.11	20.60
Spectrum 5	13.30	0.73	66.59	19.38
Spectrum 6	11.33	0.57	67.33	20.76

Figure S4. Results of EDX analysis of samples $Cs_{2+x}Sn_{1-x}Sb_xI_6$ (where x=0 – 0.04) (CS1series, part 1).

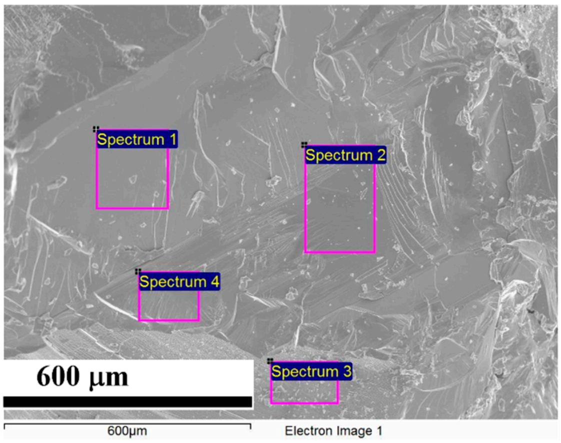

X = 0.06

	Sn	Sb	I	Cs
Spectrum 1	10.81	0.99	67.16	21.03
Spectrum 2	10.55	1.10	67.30	21.04
Spectrum 3	10.32	1.61	67.46	20.61
Spectrum 4	10.75	1.06	67.53	20.67

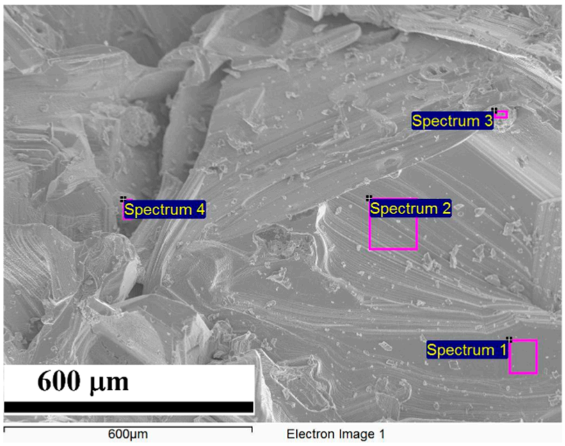

X = 0.08

	Sn	Sb	I	Cs
Spectrum 1	10.47	1.06	68.04	20.43
Spectrum 2	10.65	0.62	68.46	20.27
Spectrum 3	10.94	0.68	68.43	19.95

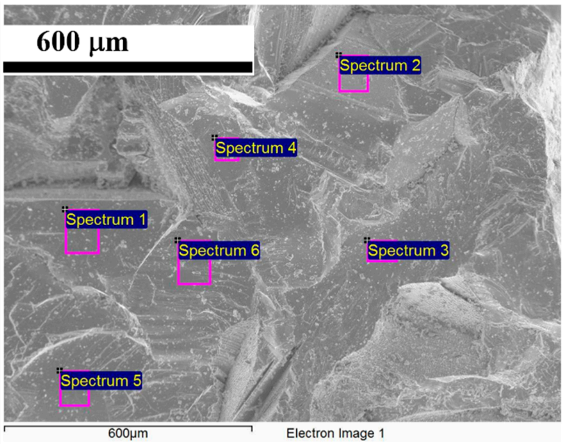

X = 0.10

	Sn	Sb	I	Cs
Spectrum 1	10.40	1.47	67.49	20.64
Spectrum 3	10.50	0.70	68.57	20.23
Spectrum 4	10.54	1.18	67.36	20.92
Spectrum 5	10.65	0.88	67.64	20.84
Spectrum 6	10.27	1.29	67.44	21.00

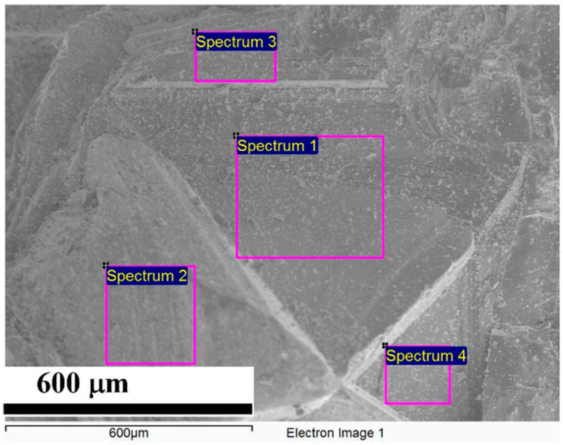

X = 0.12

	Sn	Sb	I	Cs
Spectrum 2	9.94	1.32	68.41	20.34
Spectrum 3	10.33	1.31	67.61	20.75

Figure S5. Results of EDX analysis of samples $Cs_{2+x}Sn_{1-x}Sb_xI_6$ (where x=0.06 – 0.12) (CS1 series, part 2).

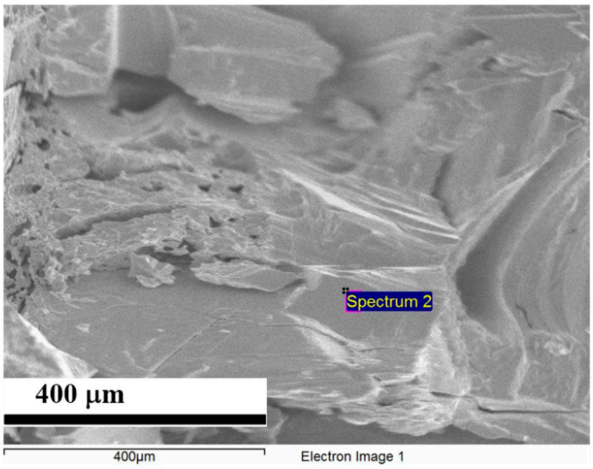

X = 0.04

	Sn	Sb	I	Cs
Spectrum 1	11.29	1.27	67.03	20.41

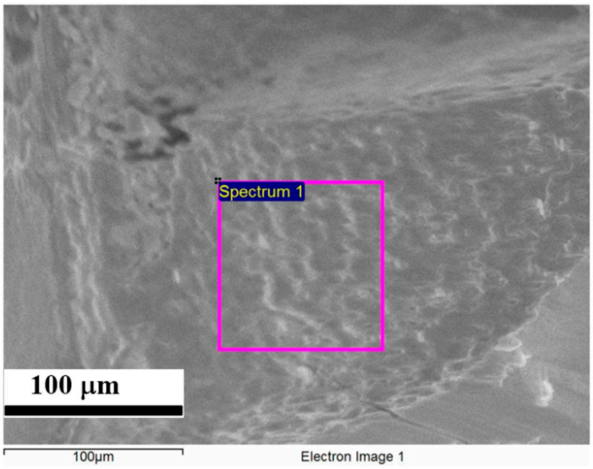

	Sn	Sb	I	Cs
Spectrum 1	10.82	0.25	67.53	21.40

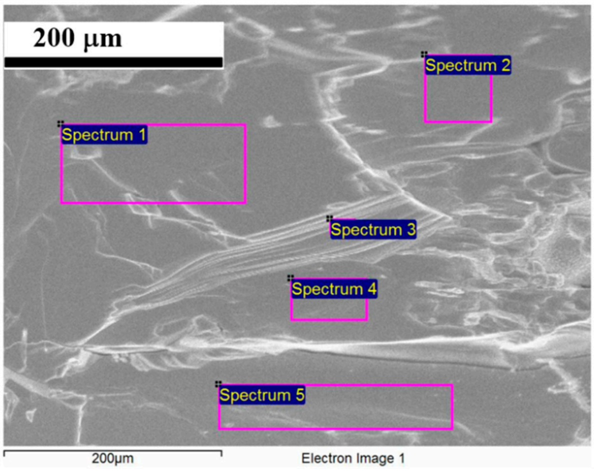

X = 0.10

	Sn	Sb	I	Cs
Spectrum 1	11.18	0.23	67.94	20.65
Spectrum 2	10.27	0.92	67.69	21.12
Spectrum 3	10.66	1.11	67.35	20.89
Spectrum 4	10.83	0.83	67.26	21.08
Spectrum 5	10.16	0.75	69.11	19.98

Figure S6. Results of EDX analysis of samples $Cs_2Sn_{1-x}Sb_xI_{6-x}$ (where x=0.04 – 0.10) (CS2 series).

X = 0.04

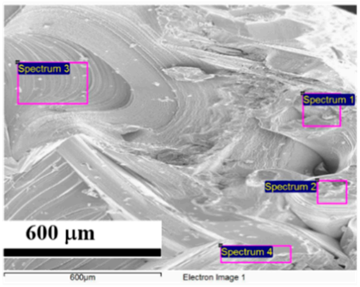

	Sn	Sb	I	Cs
Spectrum 1	8.78	3.07	67.70	20.45
Spectrum 2	10.28	0.76	68.92	20.04
Spectrum 3	10.56	0.65	67.71	21.08
Spectrum 4	10.43	0.75	68.46	20.36

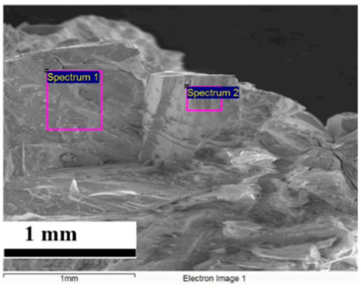

	Sn	Sb	I	Cs
Spectrum 1	10.98	0.81	67.52	20.69
Spectrum 2	10.46	0.70	67.65	21.19

X = 0.07

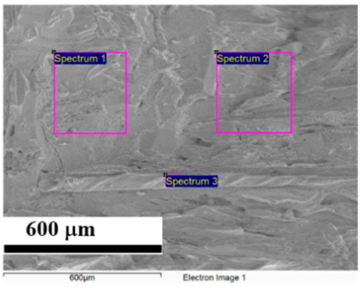

	Sn	Sb	I	Cs
Spectrum 1	10.07	1.22	68.09	20.61
Spectrum 2	10.42	0.88	67.99	20.71
Spectrum 3	10.93	0.78	67.56	20.73

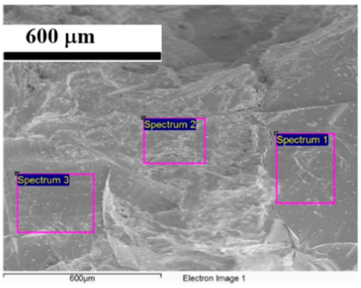

	Sn	Sb	I	Cs
Spectrum 1	10.51	0.69	67.79	21.01
Spectrum 2	9.61	1.05	68.63	20.71
Spectrum 3	10.45	1.11	67.50	20.94

X = 0.10

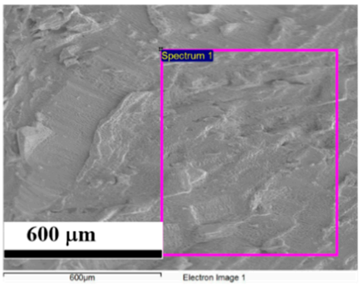

	Sn	Sb	I	Cs
Spectrum 1	9.68	1.65	68.51	20.17

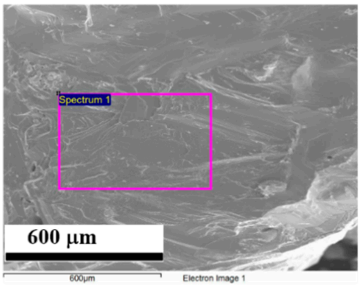

	Sn	Sb	I	Cs
Spectrum 1	10.48	1.08	67.01	21.42

Figure S7. Results of EDX analysis of samples $Cs_{2+x}Sn_{1-x}Sb_{2x}I_{6+3x}$ (where x=0.04 – 0.10) (CS3 series).

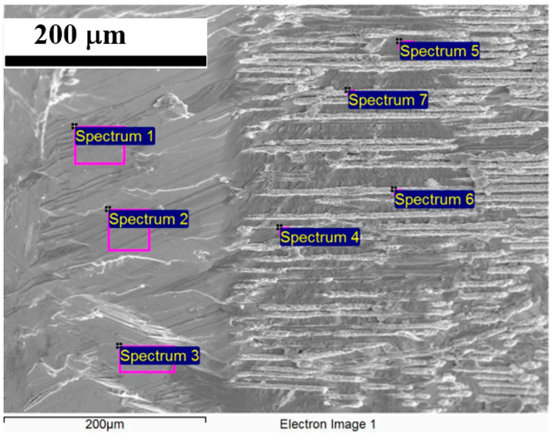

X = 0.04

	Sn	Sb	I	Cs
Spectrum 1	10.04	0.39	69.09	20.48
Spectrum 2	10.28	0.91	68.59	20.22
Spectrum 3	10.53	0.61	67.93	20.93
Spectrum 4	9.78	0.69	70.18	19.36
Spectrum 5	10.31	0.84	68.28	20.57
Spectrum 6	9.65	0.15	69.21	20.99
Spectrum 7	9.73	0.34	69.23	20.70

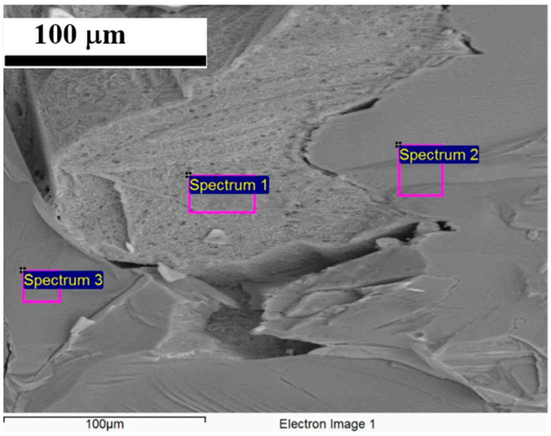

	Sn	Sb	I	Cs
Spectrum 1	7.54	0.08	70.88	21.51
Spectrum 2	10.69	0.89	67.54	20.89
Spectrum 3	10.85	1.07	67.63	20.46

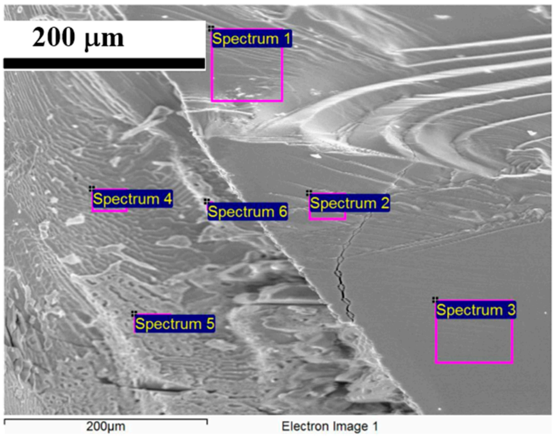

X = 0.07

	Sn	Sb	I	Cs
Spectrum 1	9.89	0.87	69.30	19.95
Spectrum 2	10.35	1.34	67.63	20.67
Spectrum 3	10.45	0.92	67.72	20.92
Spectrum 4	9.21	2.28	68.33	20.19
Spectrum 5	8.48	1.72	69.46	20.33
Spectrum 6	10.25	1.75	67.31	20.69

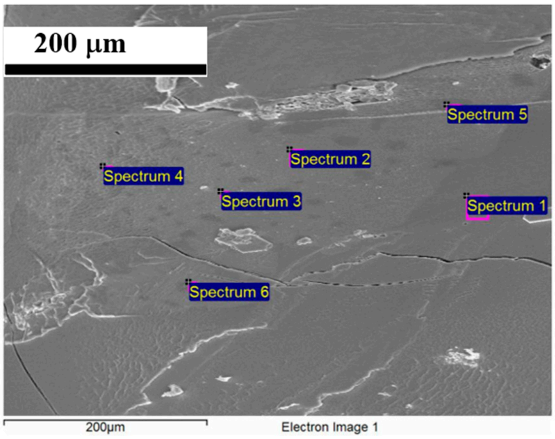

X = 0.10

	Sn	Sb	I	Cs
Spectrum 1	10.84	0.67	67.40	21.09
Spectrum 2	10.67	0.73	67.97	20.63
Spectrum 3	10.84	1.00	67.22	20.94
Spectrum 4	10.72	0.65	67.82	20.81
Spectrum 5	10.10	0.69	68.96	20.25
Spectrum 6	10.87	0.73	67.38	21.03

Figure S8. Results of EDX analysis of samples $Cs_{2-x}Sn_{1-x}Sb_xI_{6-2x}$ (where x=0.04 – 0.10) (CS4 series).

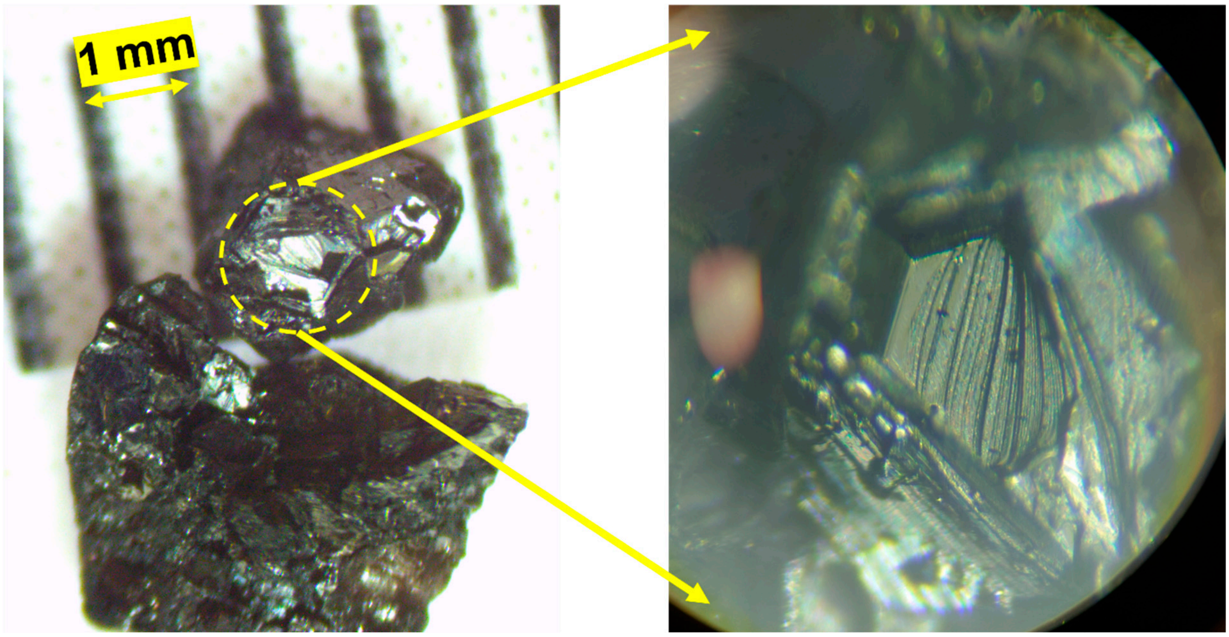

Figure S9. Optical photo of the sample x=0.08 (CS1 series).
